# Identification of Odorant-Receptor Interactions by Global Mapping of the Human Odorome

**DOI:** 10.1371/journal.pone.0093037

**Published:** 2014-04-02

**Authors:** Karine Audouze, Anne Tromelin, Anne Marie Le Bon, Christine Belloir, Rasmus Koefoed Petersen, Karsten Kristiansen, Søren Brunak, Olivier Taboureau

**Affiliations:** 1 Center for Biological Sequence Analysis, Department of Systems Biology, Technical University of Denmark, Lyngby, Denmark; 2 Centre des Sciences du Goût et de l'Alimentation, UMR6265 CNRS, UMR1324 INRA, Bourgogne University, Dijon, France; 3 Department of Biology, University of Copenhagen, Copenhagen, Denmark; 4 INSERM UMR-S973, Molecules Thérapeutiques In Silico, Paris Diderot University, Paris, France; Heidelberg University, Germany

## Abstract

The human olfactory system recognizes a broad spectrum of odorants using approximately 400 different olfactory receptors (hORs). Although significant improvements of heterologous expression systems used to study interactions between ORs and odorant molecules have been made, screening the olfactory repertoire of hORs remains a tremendous challenge. We therefore developed a chemical systems level approach based on protein-protein association network to investigate novel hOR-odorant relationships. Using this new approach, we proposed and validated new bioactivities for odorant molecules and OR2W1, OR51E1 and OR5P3. As it remains largely unknown how human perception of odorants influence or prevent diseases, we also developed an odorant-protein matrix to explore global relationships between chemicals, biological targets and disease susceptibilities. We successfully experimentally demonstrated interactions between odorants and the cannabinoid receptor 1 (CB1) and the peroxisome proliferator-activated receptor gamma (PPARγ). Overall, these results illustrate the potential of integrative systems chemical biology to explore the impact of odorant molecules on human health, *i.e.* human odorome.

## Introduction

Commonly present in food, fragrance and cosmetic products, odorants are volatile molecules that stimulate G-protein-coupled olfactory receptors (ORs) located in the olfactory sensory neurons of the nasal epithelium [1], [2]. In human, it is estimated that thousands of odorant molecules are recognized by around 400 different hORs [3]. Several studies have attempted to connect odorant physicochemical properties to the olfactory perception; however, odor coding remains largely unknown [4]–[9]. To recognize odorant molecules, the olfactory system uses combinatorial coding scheme to encode odor identities by different combinations of ORs [10], [11]. Indeed, it has been shown that one odorant can interact with several different ORs and one OR can be activated by a number of molecules. Although recent optimizations in functional expression of ORs for the screening of odorant compound libraries have been made [12], investigating all combinations is still expensive, time consuming and remains therefore a tremendous challenge. Up to now, only a small number of experimental studies have identified odorant-OR interactions in various organisms, mainly in mammals and insects [13]–[19]. Despite some efforts to elucidate the link between activation of ORs and odor perception, our understanding of peripheral olfactory coding in mammals remains limited [11], [20]–[24].

Odorant molecules might, apart from their conventional and primary role in olfaction, also trigger drug-target proteins relevant in pharmacology. For instance, studies have suggested that odor perception is involved in pathologies related to psychiatric disorders as well as in food intake behavior [25], [26]. Recently, a direct functional link between the olfactory and hormonal systems in humans has been reported [27]. Although promising, these studies remain confined to a few molecules and to a limited number of protein targets.

With the availability of large-scale chemical bioactivity databases and the recent advances in chemoinformatics and bioinformatics, it has become possible to include the chemical space in systems biology, *i.e.* systems chemical biology [28]. In addition, the application of global pharmacology profiles and network pharmacology of small molecules is emerging as a new paradigm in drug discovery [29]. However, these concepts of multi targeting have primarily been implemented for drugs [30] but not in the context of environmental chemicals. This prompted us to investigate the global network pharmacology of odorant molecules, in addition to peculiar associations between odorant molecules and hORs. In this study, we chose the term “odorome” to refer to the interactions of odorant molecules with biological targets taken as a whole.

We considered two fundamental challenges: the need for a predictive method able to decipher the peripheral olfactory coding in humans, and the potential pharmacological implications associated with odorants. Therefore, based on a newly established systems biology procedure [31], a specific human protein-protein association network (*i.e.* OR-OR network) linking ORs and odorant molecules was developed, allowing for the discovery of new odorant-OR interactions which subsequently may be tested. New suggested interactions were confirmed experimentally for six compounds and three human ORs. Further, we investigated human diseases associated to ORs by integrating a high confidence human interactome [32], [33] in the protein-protein association network developed in this context. It revealed several new functional proteins and biological pathways influenced by odorants. Lastly, we explored the potential pharmacological space of odorant compounds based on a large chemogenomics database.

From the chemical structure of a large collection of odorant molecules, annotations and predictions of the activity profile against most known biological targets were gathered. The previously unknown activity for two sets of three odorants was evaluated and confirmed experimentally for the cannabinoid receptor 1 (CB1) and for the peroxisome proliferator-activated receptor gamma (PPARγ). Thus, taking advantage of recent progress in computational chemical biology, we are able to propose new interactions, which are important for the understanding of the olfactory perception mechanism and – at the same time – highlight targets and pathways recognized by odorant compounds.

## Materials and Methods

### Odorant molecules data

We extracted 2,927 compounds, their chemical structures and their respective flavor, odor or aroma descriptions from Flavor-Base (FLB) version 2004 (http://www.leffingwell.com/flavbase.htm). Flavor-Base is one of the most extensive collections of compounds related to natural and synthetic flavoring chemicals. All chemicals are listed on the U.S. Food and Drug Administration (FDA) and Flavor and Extracts Manufacturers Association (FEMA) Generally Regarded As Safe (GRAS) list. The flavor and odor descriptions provided by Dr. J. Leffingwell and Associates are also supported by several published studies sources such as Arctander [34].

All the selected molecules possess at least one odorant component described as “odor”, “flavor”, or “aroma”, excluding the molecule exclusively described as “taste”. Existing variations in organoleptic descriptions by various authors were taken into consideration. Note that no information about interactions between odorant molecules and ORs is provided in Flavor-Base.

For the set of compounds extracted, we compiled odorant molecule-OR binding interactions from the literature and the Olfactory Receptor Database (ORDB) [35] for human, rat and mouse (Table S1 in File S1). Only direct physical interactions were considered (*i.e.* binding data) and none of the gene expression was kept in this study.

### Human odorome

To create the human olfactory network, we developed a protein-protein association network (defined as an OR-OR network in this study). The OR-OR network was generated by initiating a node for each human OR, and by linking any OR-OR pair where at least one overlapping odorant was identified. To reduce noise and select the most significant OR-OR associations, we assigned a weighted score to each OR-OR association. The weighted score was calculated as the sum of weights for shared odorant molecules, where weights are inversely proportional to the number of associated ORs for a given odorant as previously described and thoroughly benchmarked against two gold standard repositories [31]. The resulting human OR-OR associations network contains 24 ORs connected via 463 associations.

In a second step, the human olfactory network was enriched with rat and mouse odorant molecule-OR binding interactions gathered previously. To do so, the non-human OR names were translated into their human orthologous genes using YOGY [36] For ORs that have no orthologous human gene, homology searches were performed using BLASTP [37]. Human ORs with the highest score and E-value associated to rat or mouse ORs were integrated in the olfactory network represented by human odorant-OR interactions. All OR names were converted to Gene ID using UniProt [38]. In total, 83 ORs and 323 molecules with binding information to at least one OR were collected and integrated in the OR-OR network resulting in 938 unique associations. It is important to notice that the discrimination between ORs agonist and antagonist is not included in the study and our network cannot be used to identify odorant synergies or opposite effect on ORs.

Panels of odor descriptions were also associated to the molecules using the Flavor-Base database. Therefore, we were able to retrieve 189 odor for 230 odorant molecules among 323 compounds binding to OR proteins, and to map the odor perceptions associated to chemicals in the OR-OR network.

### Integrating odor descriptions into the human odorome

To evaluate the tendency and selectivity of odors associated to ORs, we developed an association score (AS) based on the number of compounds associated to an odor. The AS is calculated using the equation: 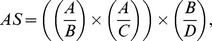
where AS is the association score, A the number of molecules for one OR, B the total number of compounds carrying one odor, C the number of ORs for the same odor, and D the total number of molecule-odor interactions. In our study D = 4193. Table 1 presents an example of the results obtained for the odor “anis”.

**Table 1:** List of ORs predicted to interact with molecules carrying the “anis” note.

Odor	ORs	Number of compounds (A)	AS
anis	OR1D2	2	1.59 10-4
anis	OR1D20	3	3.57 10-4
anis	OR1G1	5	9.93 10-4
anis	OR52D1	5	9.93 10-4
anis	OR5D18	4	6.36 -10-4
anis	OR6A2	2	1.59 10-4
Total	C = 6	B = 21	

With this formula, we can associate a score between each odor and each OR. The higher the score, the more significant is the interaction. In this example, OR1G1 and OR52D1 are the most significant association to the odor “anis”.

Results for the four highest odors associated to each human OR are shown in Table S2 in File S1.

### Predicting novel hOR target for odorant molecules

A network protein procedure was generated to predict interaction between hOR and odorants using the developed human odorome. This network-neighbor's pull down approach is a three steps procedure: (a) selection of the input hORs: extraction of the hORs known to be associated with the selected odorant molecules from the available literature information. (b) Identification of network(s) surrounding the input hORs by a neighbor protein procedure. In this procedure, our odorome was queried for the input ORs, and associations between them were compiled. For each neighbor, a score was calculated taking into account the topology of the surrounding network, based on the ratio between total interactions and interactions with input ORs. (c) Establishment of a confidence score for each OR: each of the pull down complexes was tested for enrichment on our input set by comparing them against 1.0e4 random complexes for OR-OR association set to establish a score for each connected OR. The score was used to rank ORs to select potential hORs targets for odorants. The accuracy of this procedure was demonstrated previously for known drugs and drug targets [31].

### Integrating the human interactome and the odorome

Protein-protein interactions (PPIs) were extracted from a list of ORs and their first interactor proteins using an in-house human interactome network based on experimental data from human and model organisms [32], [33]. The current interactome contains 507,142 unique PPIs linking 14,441 human proteins. PPIs of the 83 ORs allowed extending the odorome to 183 genes. This network was used for the disease and pathways enrichment analysis. Human disease information was extracted from the GeneCards database [39]. We also determined the enriched terms among pathways using the KEGG and Reactome databases. Protein-disease relationships and gene-pathway links were independently evaluated in the odorome. P-values were calculated using hypergeometric testing with Bonferroni adjustment for multiple testing [40]. Results are shown in Table S3 in File S1.

### Odor-target matrix

A chemogenomic database, ChemProt, was used to explore the human pharmacological space with the FlavorBase odorant compounds. ChemProt is a chemical genomics platform that integrates chemical-protein interactions from various available data sources [41]. The current version of ChemProt as of January 2013 contains 1,150,000 unique chemical structures with biological information for more than 15,290 proteins [42]. We considered only compounds with binding activity in this study.

### Mapping odorant molecules in the pharmacological space

Each chemical structure from FLB and ChemProt was encoded into binary strings using the Molecular ACCess Systems keys (MACCs) to investigate structural similarity between FLB compounds and ChemProt chemicals. Using the Tanimoto coefficient (Tc), the degree of similarity between two molecules was quantified. Chemical-compound networks were generated to visually display compounds from FLB having a high similarity coefficient with ChemProt molecules using Cytoscape [43].

### GloSensor cAMP assay

To validate predicted interactions between odorant compounds and ORs or the CB1 receptor, we used the GloSensor cAMP assay from Promega and measured the EC_50_ values of compounds. This luminescent assay is a sensitive method for measuring Gs and Gi- protein coupled receptor activation by real-time detection of intracellular second messenger cAMP [44]. The protocol is described in the GloSensor cAMP assays paragraph in the Supplementary Methods (S_file).

### Competitive PPARγ binding assay and trans-activation assay

To validate predictions of odorant-PPARγ interactions, IC_50_ values for respective compounds were determined by competitive binding using time-resolved fluorescence resonance energy transfer (LanthaScreen, Invitrogen) on a Wallac EnVision (PerkinElmer). Furthermore, to assess the bioactivities of the predicted molecules, a PPARγ lipid-binding trans-activation assay was used (PPAR LBD). The protocols are described in details in Competitive PPARγ binding assay and PPAR-LBD Transactivation paragraphs in the Supplementary Methods (S_file).

## Results

**Figure 1:**
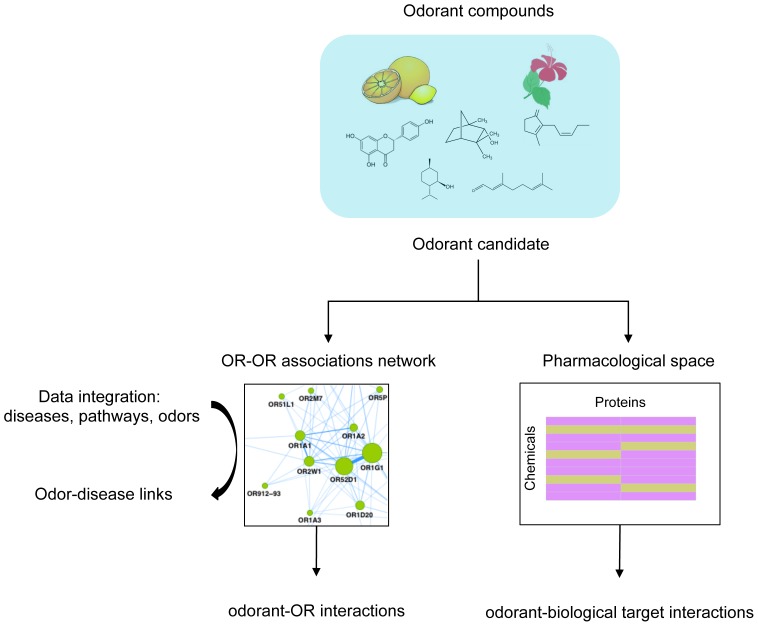
Workflow of the study. Strategy to improve knowledge of olfactory perception and biological roles of odorant molecules. First an OR-OR association network identifies novel odorant-OR interactions for odorant candidates. Second, pathways linked to proteins are integrated in the OR-OR network allowing deciphering odor-disease connections. The last step involves scoring and ranking of odorant candidates for biological targets within the pharmacological space.

To improve knowledge of olfactory perception and biological roles of odors in human, odorant molecules were used to generate a predictive model to identify odor coding, and to explore the known pharmacological space. We integrated various data type such high confidence protein-protein interactions and large chemical biology database to underlie molecular mechanisms of odorant molecules and the biological pathways they perturb. Overall, the results show a global mapping of the human odorome. The key steps of our approach are illustrated in Figure 1.

### Modeling of the odorant human combinatorial coding

#### Generation of a human odorome

**Figure 2:**
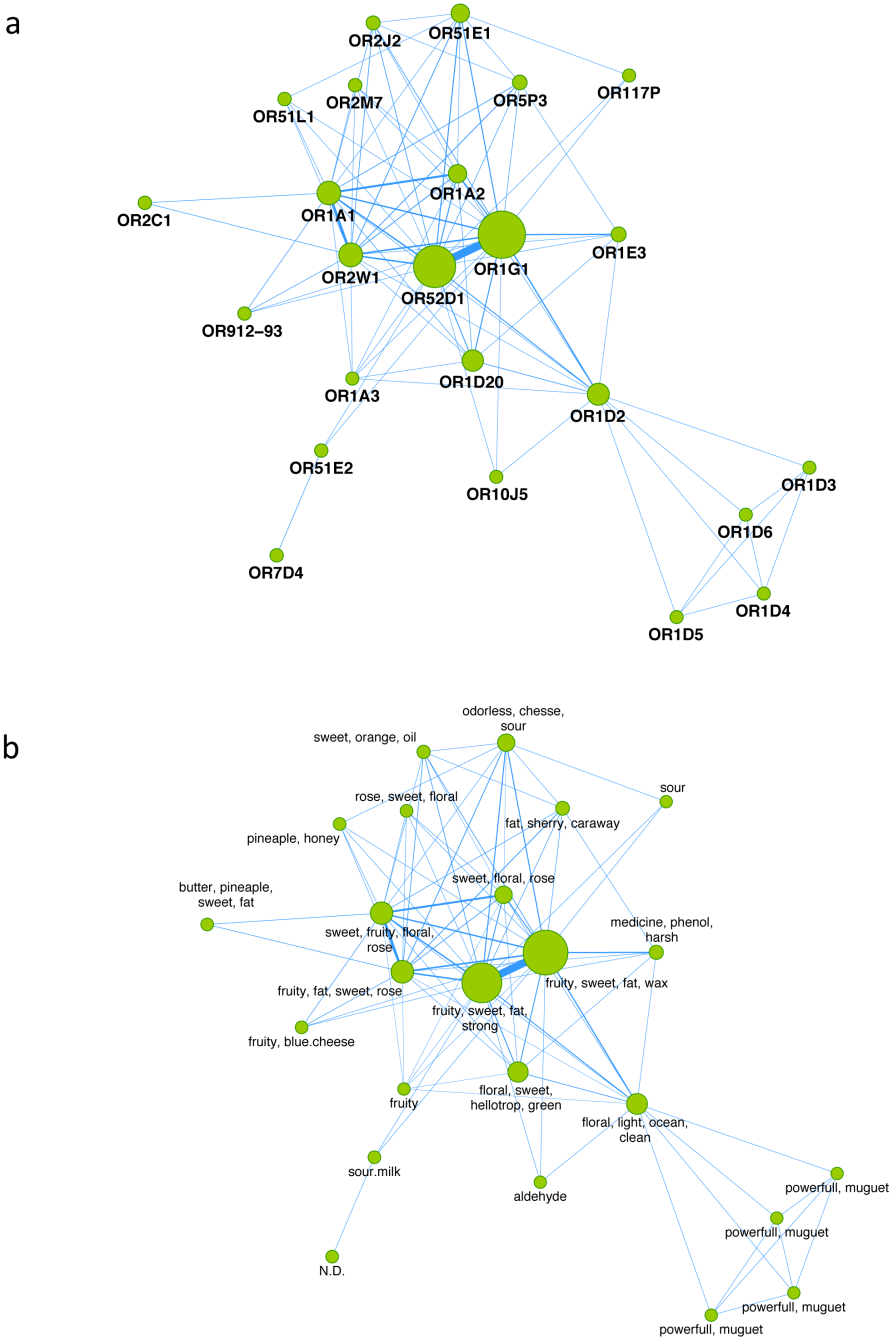
View and mapping of the odor in the OR-OR association. (a) View of the human odorome. Nodes and edges represent the human ORs and the connections between the ORs, respectively. The node size corresponds to the number of odorant molecules known to bind to a particular OR. A weighted score, represented by the width of the edges, was assigned to each OR-OR association. It represents the strength of the link between two ORs as defined by the number of shared compounds for both ORs. (b) Mapping of odor descriptions on the human odorome using the association score (AS). Odor(s) tendency for ORs were integrated into the human odorome map. (N.D. =  non-determined odor for OR7D4).

To explore the organization of the odor space in humans, i.e. how ORs respond to an odorant, we compiled from the literature a list of carefully curated chemical-OR interactions from human (Table S1 in File S1). In total, we gathered 189 odorant molecules associated to 24 human ORs through 463 interactions. We implemented the “target hopping” concept i.e. if two proteins both bind to the same ligand, they can be considered as interacting in the same chemical space [45]. So, assuming that two ORs biologically activated with the same molecule are likely to be involved in a common mechanism of stimulation, we developed a protein-protein association network for ORs (defined as an OR-OR network) in a similar manner as described previously [31]. The OR-OR network, depicted in Figure 2a, clearly shows that some ORs are highly connected such as OR52D1 and OR1G1, whereas other ORs are sensitive to very specific molecules only.

In addition, from the OR-OR network, we mapped the odor perceptions associated to the chemicals (Figure 2b) integrating the information from Flavor-Base and ORDB. Studies have reported that chemicals having a similar odor profile may activate the same receptors [11], [23], [46]. However, in our compilation the majority of chemicals have multiple annotations with several odors e.g. dihydrojasmone has fresh, fruity, jasmine and wood odors. Using an association score (AS), we prioritized ORs to odors and identified odor tendencies for a given receptor (for odor-OR relationships see Table S2 in File S1). For example, our approach depicts that OR1G1 is highly stimulated by fatty and waxy notes [46]. Some general notes *e.g.* “fruity” appear to be connected to many receptors. In opposite, quite few notes are linked to only one OR *i.e.* “light”, “ocean” and “clean” are related to OR1D2 and “medicine” and “phenol” are linked to OR1E3.

From the network, we can identify also hubs of ORs that are more related to a given odor. For example, “muguet”, a floral odor, is exclusively reported to OR1D3, OR1D4, OR1D5 and OR1D6, and “sour” odor is associated to OR51E1, OR117P and OR51E2. Interestingly, although “pineapple” is associated to OR51L1 and OR2C1, there is no connection between the two ORs. In fact this OR-odor association comes from different compounds that have not been tested on the same OR. Obviously, the method is dependent of the diverse experiments performed so far on ORs and reflects that some ORs have been tested more than others. However, the network provides a global visualization of the human odorome based on current knowledge.

#### Deciphering novel odorant molecule- hOR interactions

**Figure 3:**
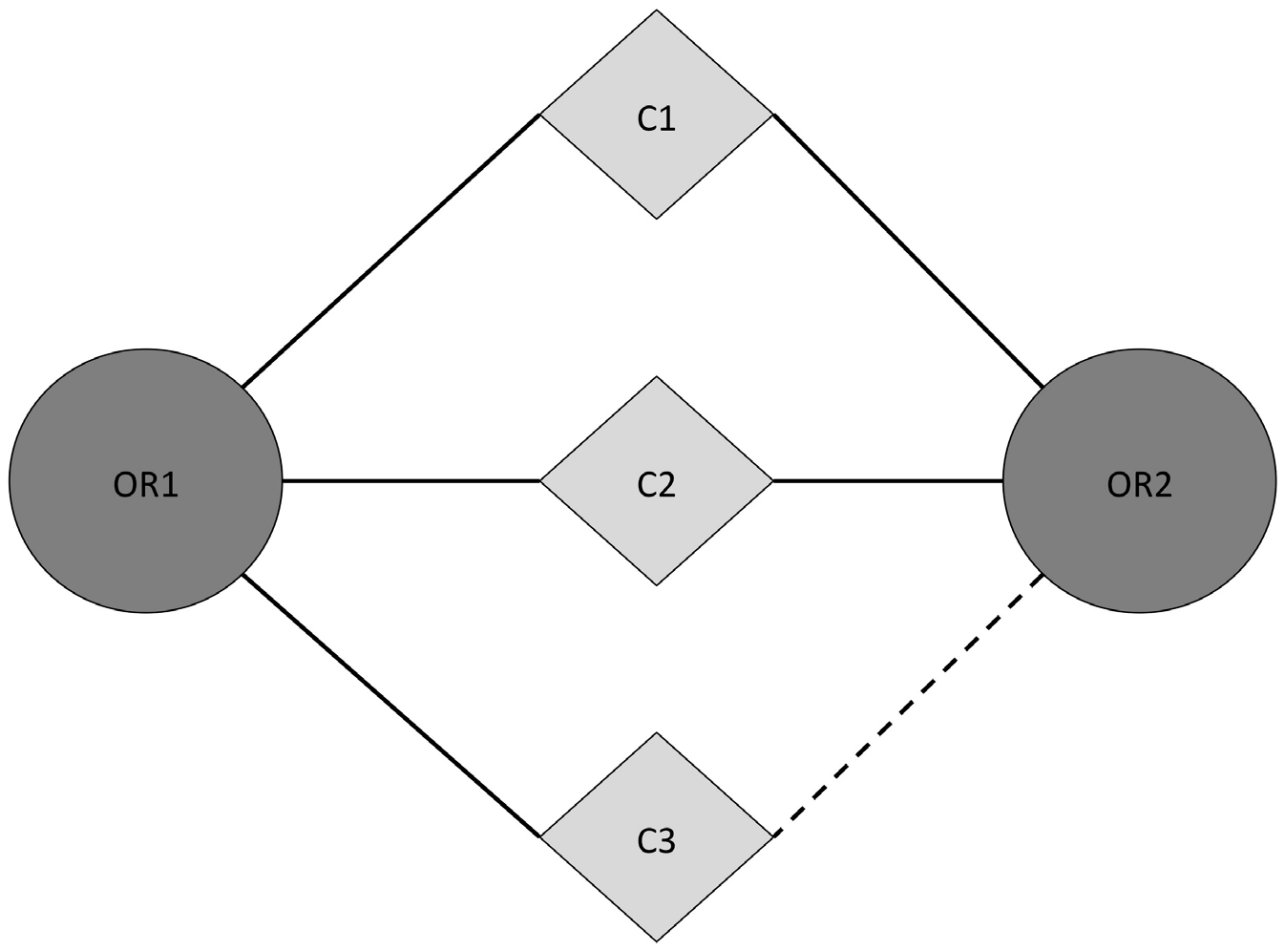
Schema of the OR-odorant prediction concept. In this example, C3 is an odorant predicted to bind to OR2 because is binding to OR1 like C1 and C2.

An interesting aspect from the OR-OR network is the possibility to suggest new odorant-OR interactions that were not studied previously. Based on the assumption that if two ORs are affected by two odorants, and one of the OR is further deregulated by an additional odorant, it might be that both ORs are in fact affected with the same three odorants as shown in Figure 3. Using a neighbor protein procedure, an association score between each OR and each odorant can be computed, as described previously [31], [32]. From, the developed network it is then possible to evaluate the significance of the odorant-OR association as well as to predict the association for new ligand-OR.

To assess the performance of our approach, we decided to test a set of compounds experimentally. As we had bioassays for OR2W1, OR5P3 and OR51E1, we focused on these 3 ORs for the validation.

**Figure 4:**
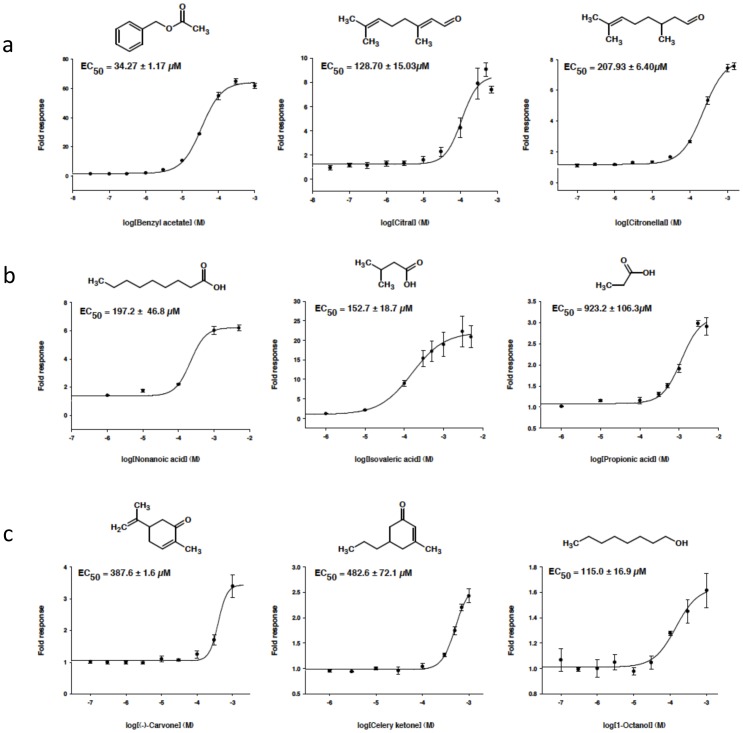
Concentration-response curves of odorants for human ORs. Odorants predicted as agonists ( =  predicted compounds) and odorants previously shown to be agonists by Saito et al. 2009 (positive controls) activated four human ORs: (a) OR2W1, (b) OR51E1 (c) OR5P3. Data points and EC_50_ values are means ± s.e.m. from at least three experiments.

Citral and Citronellal, two compounds naturally produced in the oil of various plants including lemongrass and orange, have been shown to be strong agonists of OR1A1 [47]. These compounds were reported to be also ligands of OR1A2 [47], [48]. Based on the OR-OR network, these compounds show a strong association score with these ORs but also may interact with OR2W1 (Table 2). As the stimulation of OR2W1 by these two compounds was not reported in the literature, we decided to test this prediction experimentally using OR-transfected Hana3A cells and a functional assay adapted to GPCR screening, the GloSensor cAMP assay [44]. Citral and citronellal were found to activate OR2W1 with EC_50_ values of 128.7 μM and 207.9 μM, respectively (Fig. 4a). These odorants were about 4 to 6 fold less efficient than benzylacetate, one of the best OR2W1 ligands (EC_50_ = 34.7 μM) [13].

**Table 2:** Prediction of novel OR-odorant interactions.

Odorant	Known OR	Score*	Predicted OR	Score*
Citral	OR1G1	0.171	OR2W1	0.889
	OR52D1	0.171		
	OR1A1	0.358		
	OR1A2	0.748		
Citronellal	OR1G1	0.171	OR2W1	0.889
	OR52D1	0.171		
	OR1A1	0.358		
	OR1A2	0.748		
1-octanol	OR1G1	0.512	OR5P3	3.458
	OR52D1	0.512		
	OR2W1	0.826		
	OR1A1	1.013		
	OR1A2	2.051		
	OR51E1	2.425		
	OR2J2	3.458		
Celeryketone	OR1G1	0.171	OR5P3	1.225
	OR2W1	0.287		
	OR1A1	0.358		
	OR1A2	0.748		
Isovaleric acid	OR1G1	0.171	OR51E1	0.883
	OR52D1	0.171		
	OR117P	2.704		
Propionic acid	OR1G1	0.093	OR51E1	0.511
	OR52D1	0.093		
	OR51E2	1.501		

* To find ORs interacting to odorants, a neighbor protein procedure was used which score the association between ORs and odorants. The lower is the score, the stronger is the association.

We also investigated the activation of other ORs by new compounds (Figs. 4b, 4c). For example, from our OR-OR network, we predicted that two new compounds, 1-octanol and celery ketone (two OR1G1 ligands) might interact to OR5P3 (Table 2). Experimentally, we observed that both compounds activate this receptor with EC_50_ values of 115 μM and 482.6 μM respectively, which indicates that these odorants are as active as (-)-carvone (EC_50_ = 387.6 μM), a known OR5P3 ligand [13]. Similarly, isovaleric acid and propionic acid (OR1G1 and OR52D1 ligands) were identified as new putative ligands of OR51E1 (Table 2) and tested experimentally. Isovaleric acid activated OR51E1 in the same range as that observed for nonanoic acid, a known ligand of this receptor (EC_50_ = 152.7 μM and EC_50_ = 197.2 μM, respectively) [13]. Conversely, propionic acid showed an activity 5 fold lower with an EC_50_ = 923.2 μM. Finally, we should notice that the tested compounds did not induce any response in mock-transfected cells. However, for some of the compounds, *e.g.* celery ketone and 1-octanol, no saturation could be observed because of cytotoxic effects at concentrations higher than 10^−3^ M.

### Linking the human odorome to diseases and pathways

To investigate dysfunctions and diseases associated with the olfactory system, we enriched the developed OR-OR network by integrating data gathered from mouse and rat (Table S1 in File S1). Although the odor perception from one species to another one might be different, OR orthologs tend to show conserved ligand interactions [49].

ORs from rodents were linked to human orthologs resulting in a total of 775 additional chemical-protein interactions (Table S4 in File S1). Consequently, the new OR-OR network contained 938 interactions between 83 proteins (Fig. S1). We then integrated protein-protein interactions (PPIs) into the human odorome, and constructed a PPI network for the set of 83 human ORs. The interactome used was a high confidence set of experimental PPIs extracted from a compilation of diverse data sources [32], [33]. A total of 183 new genes were identified and among them, 12 were connected to at least one of the 83 ORs with high confidence scores. In general, ORs appear to interact with three guanine nucleotide binding proteins: GNAL, GNGT1 and GNB1. OR1G1 is linked to a fourth protein, the odorant binding protein 2B (OBP2B). In a second step, disease enrichment data were included with the aim of prioritizing disease candidate genes. Among them, three disease groups are statistically significantly connected: hypertension, schizophrenia and mood disorders (list of genes and p-values can be found in Table S3 in File S1). A previous study has explored the potential of olfactory dysfunction as a key component in early diagnostic strategies of Parkinson and Alzheimer diseases [50]. The UPSIT (University of Pennsylvania Smell Identification Test) revealed abnormality more frequently for patients with neurological diseases than olfactory-evoked responses (http://emedicine.medscape.com/article/861242-overview). For hypertension, patients with smell impairment are reported to use larger quantities of sugar and salt to highlight flavors and thus increase the risk of developing hypertension [51].

We analyzed also the functional properties of the olfactory system using two pathways repositories (KEGG and Reactome) [52], [53]. From the KEGG database, we observed statistical significance for the calcium signaling pathway, the neuroactive ligand-receptor interaction pathway, the taste transduction pathway, the type 2 diabetes pathway and the long term depression pathway. From the Reactome database, three pathways were significantly linked to the global odorome, the ‘opioid signaling’, the ‘integration of energy metabolism’ and the ‘GPCR signaling’ pathways.

A recent study supports our findings by showing that odor-identification deficit and memory impairment are closely associated with disease-specific metabolic changes [54]. Similarly, it is speculated that flavor molecules are suggested to play a role in food intake and thus potentially increase prevalence of overweight and obesity [55]. A possible mechanism to reduce food intake could involve a perturbation of the opioid signaling pathways.

Overall, these biological networks revealed interesting functional properties and biological pathways involving known drug-targets. Such results imply that odorant molecules interact not only with ORs but might also affect drug-targets.

### Mapping the odorant pharmacological space

As a final step we investigated all biological targets potentially recognized by odorant compounds. Chemical-protein interactions for a complete biological system are usually unknown apart for some drugs, and the majority of molecules have only been studied for one or few protein targets. This is especially true in the case of odorant compounds, which have been mostly studied on ORs.

We decided to identify potential novel and unexpected odorant-protein interactions. Assuming that chemicals sharing highly similar structure also share similar biological properties [56], we used ChemProt, a large curated chemogenomic database of more than 1 150,000 molecules with over two millions chemical-protein interactions [41]. Using MACCs fingerprints and a strict Tanimoto coefficient (Tc) distance threshold of 0.9, 1,091 odorants were identified with odorant-protein interactions for 821 proteins. Interestingly, for 329 odorants, links to 200 proteins were already available (i.e. compounds tested for a protein), represented by 556 unique chemical-protein interactions. For example, capsaicin, described in Flavor-Base with a “slight herbaceous odor” (known also for its strongly perceived “burning hot pungent taste”) has shown agonist activity on the human vanilloid receptor 1 (TRPV1) and inhibition of PTGS1, a well-known protein inhibited by non-steroidal anti-inflammatory drugs such as aspirin. Another example, thymol is activating a human transient receptor (TRPA1) which has a central role in the pain response to endogenous inflammatory mediators [57]. In order to reveal common structural features between odorants and other molecules, we visualized their distribution inside the chemogenomic database by developing a chemical similarity network, excluding annotations (Fig. S2). The generated network can be interpreted in two ways: some odors (green) form large clusters, which appear to share similar features with few chemicals from ChemProt (blue). For instance, 2-heptyl-butyrate (wax, fruit, green, tropical, floral) is structurally similar (Tc of 0.952) to the fatty acid isopropyl palmitate, a solvent for fragrance agents and known to have binding affinity to the human cannabinoid receptor 2 (CB2) [58]. Other odorants show structural similarity with molecules possessing large set of bioactivities in ChemProt. This is the case with theobromine, a cosmetic additive, similar to caffeine and theophylline, two compounds intensively studied in healthcare [59].

Based on Uniprot identifiers, the 821 proteins potentially targeted by odorants were categorized into 285 families, and 26 of them have more than 100 interactions with odorant molecules (Fig. S3). Not surprisingly, the G-protein coupled receptor family (GPCR), which contains ORs, is the most common type predicted. A majority of odorant-GPCR associations are with cannabinoid receptors (69%). Few other predictions are for metabotropic glutamate receptors (2%) and opioid receptors (1%). Ligand-gated ionic channel, amidase enzyme family, nuclear receptors and cytochrome P450s are also families largely targeted by odorants which is consistent with previous discovery linking the olfactory system with ligand-gated ion channels and amidase [60], [61].

**Figure 5:**
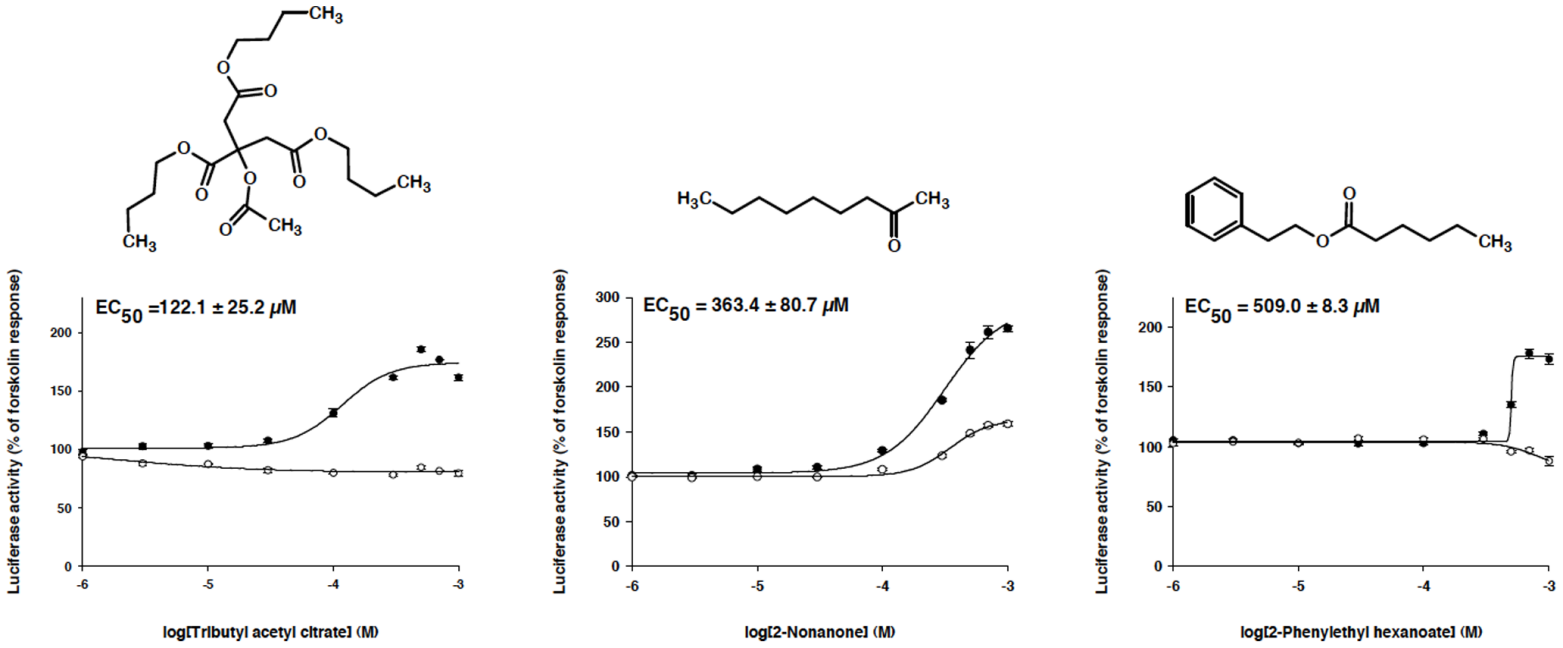
Concentration-response curves of odorants for the human cannabinoid receptor CB1. Predicted compounds, tributyl acetyl citrate, 2-nonanone and 2-phenylethyl hexanoate acted as inverse agonists. GloSensor assays were carried out in the absence (•) or in the presence (○) of pertussis toxin-treated cells. Data points and EC_50_ values are means ± s.e.m. from three experiments.

We decided to investigate the interactions of odorants with the human cannabinoid receptor CB1 using CB1-transfected HEK293 cells and the GloSensor cAMP assay, because of its large representation in predicted proteins as target, its role of endocannabinoid system in metabolic diseases [62], and its probable link with olfaction [63]. We first checked that our experimental system worked efficiently by testing cannabinoids acting as agonists (AEA, HU210) or inverse agonist (AM251) (Fig. S4). Then, amongst the molecules predicted as CB1, we selected two of them (i.e. tributyl-acetylcitrate and 2-phenylethyl hexanoate) due to their structural similarity with hexadecyl propanoate, a known inhibitor of CB1 [58]. In addition, we looked on the flexibility of the molecules able to map to the structure of anandamide, a known natural CB1 ligand [58], [61] and select the compound 2-nonanone for testing. The three compounds, tributyl-acetylcitrate, 2-nonanone and 2-phenylethyl hexanoate were found to interact with the CB1 receptor although with a weak EC_50_ (Figure 5). They elicited an increase in cAMP production in control cells, this effect being blocked in pertussis toxin-treated cells. These results indicate that the predicted compounds acted as weak inverse agonists, with EC_50_ values varying from 122 μM to 509 μM. All tested compounds did not induce any response in mock-transfected cells.

We looked also into nuclear receptors and more specifically PPARγ, a target also associated to metabolic syndrome, inflammation and type-2 diabetes. PPARγ has shown an interesting response to the application of nutrition-based interventions [64]. Indeed, it has been reported that naringenin, from grapefruit or elderflower, stimulated PPARγ transactivation making cells more sensitive to insulin [64]. PPARγ is also involved in the regulation of fatty acid storage and glucose metabolism, and it has been recognized that nutritional supplementation such as omega-3 fatty acids and polyunsaturated fatty acids influence the inflammatory response of some diseases such as inflammatory bowel disease (IBD) [65]. Moreover, PPARγ have been identified to interact with endocannabinoid system [66]. We decided therefore to investigate the interaction of odorants on the PPARγ protein.

**Figure 6:**
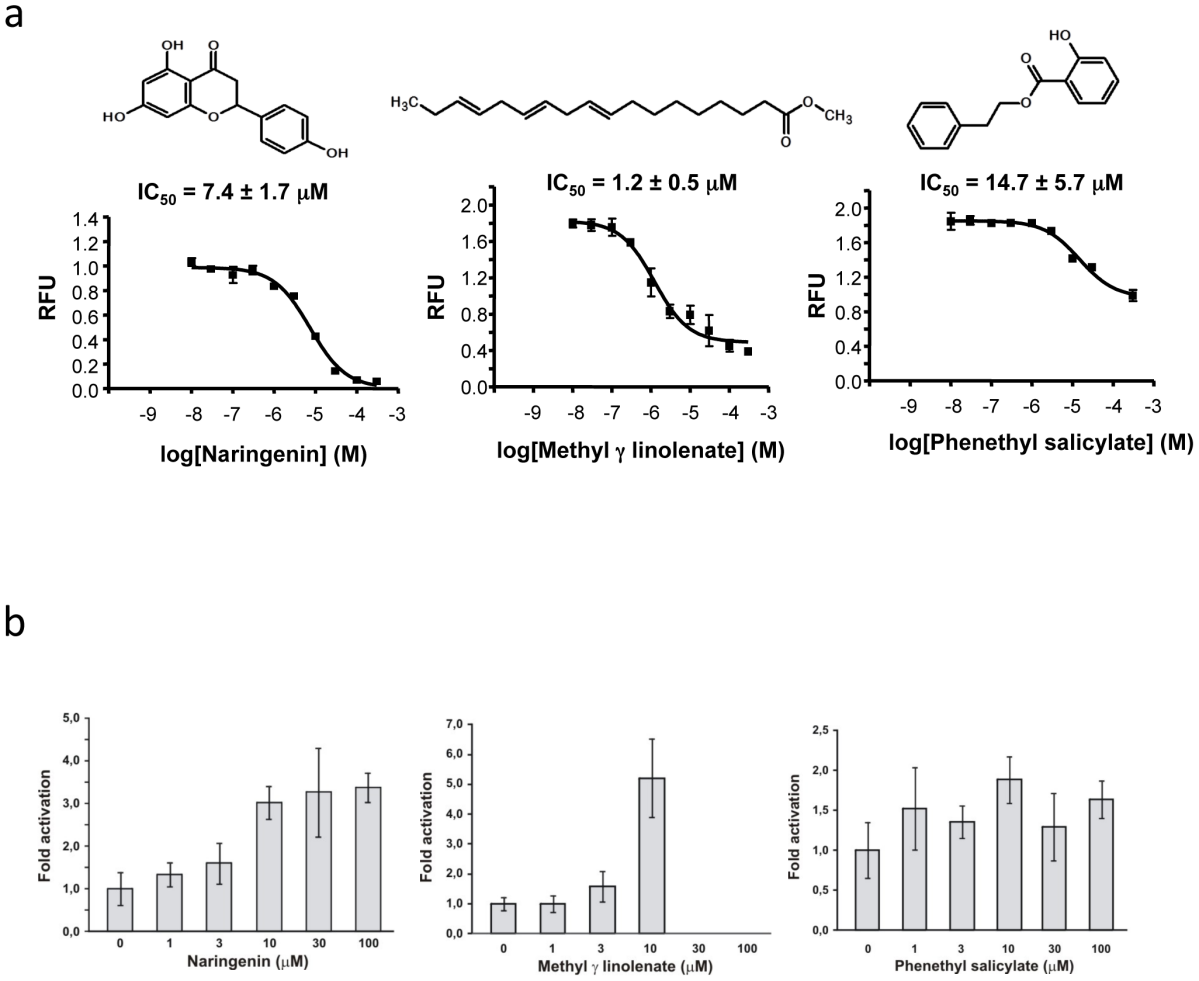
Results of bio-activation of three odorants on the PPARγ receptor. (a) Concentration dependent ligand displacement of three odorants predicted as ligands for the PPARγ receptor. (b) Transcriptional activation of PPARγ by three odorants. Results are shown as the average ± standard deviation of 2 individual experiments with each of the experiments performed with 8 replicas. Activation is given as fold activation relative to the DMSO vehicle. Rosiglitazone (not shown) was used as positive control.

Among them, we considered naringenin, methyl γ linolenate and 2-phenylethyl salycilate that show structural similarity with PPARγ ligands (kaempferol, lauric acid methyls ester and benzenepropanoic acid, 4-([1,1′-biphenyl]-2-ylmethoxy) respectively). Using a competitive PPAR binding assay, inhibition of binding (IC_50_) values for three predicted compounds were determined. Interestingly, all compounds showed binding activities on PPARγ at the μM scale, validating the multi-activities of odorants in cellular processes other than olfaction (Figure 6a). Compared to the reference molecule, rosiglitazone (IC_50_≈50 nM in this assay), naringenin shows good affinity to PPARγ (IC_50_ = 7.4 μM). The food additive phenylethyl salicylate presented activities with the same range (IC_50_ = 9.2 μM) whereas methyl γ linolenate a fatty acid compound naturally present in banana, grapefruit juice, grape, melon, strawberry, tomato and chicory have an activity on PPARγ seven fold better than naringenin (IC_50_ = 1.2 μM). To assess the biological activities of these compounds, activation of the PPARγ ligand binding domain (LBD) was investigated using trans-activation assays (see Methods and SI). As a result, all three compounds were able to activate the PPARγ-LBD although with low efficiency (Figure 6b). The apparent partial agonist properties of the compounds were reflected by the ability to partly antagonize rosiglitazone-induced transactivation in the same assay (data not shown). Overall, our findings suggest that these compounds might have interesting anti-diabetic and anti-inflammatory properties.

## Discussion

Two approaches were developed in this study in order to generate a human odorome: first, we identified some new odorant-OR interactions as well as putative pathologies and pathways associated to olfaction, and secondly, we proposed potential therapeutic properties of a number of odorant molecules.

To start, we have developed an innovative approach for predicting molecule candidates to hORs. Previous studies have extensively used molecular structures of odorants and molecular modeling to suggest such interactions [67]–[70]. The ability to make new findings is illustrated by the development of a protein-protein association network on ORs, which led to identification of new ligand-OR interactions. As all models based on experimental data, our proposed strategy is in a great dependence to the nature of available data. We collected odorant that bind to ORs from public available resources and negative control was not considered in our model, which is unidirectional. One of the limitations is, only 24 human ORs showed bioactivity, which represent only 6% of the human olfactome. Moreover we should take into consideration the so-called ‘Matthew effect’ [71] resulting in maintained research interest regarding already well-investigated odorants and ORs, and then a larger amount of available data for these odorants and ORs (OR1G1, OR52D1…). This skews the findings towards interactions involving ORs already intensely investigated (OR2W1). But this also highlights the ORs, which need further attention. Hence the lack of predicted interaction between odorants and OR5P3 for instance might be the result of less available data to create the model rather than lack of biological effect. The successful experimental validation on well-known OR (OR2W1) and less-known OR (OR5P3) show the innovative level of such computational approach.

The previous identification of targets repertoire is a crucial importance, and could be very difficult to establish, especially in the case of OR targets. Indeed, there is probably a gigantic number of odorant molecules; some authors mention about “myriad of flavors”, and Mori estimated “that more than 400 000 different compounds are odorous to the human nose” [23], [72]. In as much as a maximum of one hundred of molecules have been tested on each expressed OR, it is difficult to ensure that the best ligands of each studied OR have been identified [23]. Nevertheless, these data are now available and can be used for the development of computational approaches able to decipher the olfactory repertoire. For example, we could imagine that the integration of negative data and degree of affinity of ligands to ORs could be of great value in such OR-OR network.

From the OR-OR network, we proposed several associations between odor and ORs. It is well admitted that an odor results from the perception of a mixture of molecules. In other words, odors described for example as “strawberry”, “green” or “woody” have probably no real intrinsic existence, but report to some environmental contexts. Consequently, humans need a lot of words related to their contextual memories, to give full account of their own perception, [73]–[75]. That may explain why the odor of a molecule is rarely described by a sole odorant note but rather by several notes. Each odor described by humans could be more adequately defined as an ensemble of several “components”. Moreover, as the perception of odors results from a combinatorial coding, this implies the unlikelihood to associate strictly an odor to a sole receptor. Conversely, an OR might be associated to a component of an odor.

In addition to the OR-OR network, integration of the interactome and phenotypic data in the network allowed for a second level of prediction capability. Disease gene candidates can be prioritized, highlighting the potential role of the olfactory system as a biomarker for diseases.

The second aspect of our work was to obtain an overview of drug-targets (i.e. proteins) interacting with odorant molecules by systematic structural similarity searches using a large chemogenomic repository. Such exploration of the pharmacological space was previously reported for drug compounds [76], characterized by drug-protein associations. Up to now, no investigation has been reported in the literature regarding large set of odorants. The *in vitro* validation of predicted odorant binding to CB1 and PPARγ supports the possible pharmacological relevance of the newly identified odorant-target relationships, although further studies are necessary to gain more insight into the diffusion and the biotransformation routes of such compounds to reach these targets.

Expanding the knowledge of our sense of smell by integrating systems chemical biology of odorant molecules in drug discovery is an attractive way to move forward in the quest to identify effective drug-food combination therapies [77]. Recently, the development of an electronic nose to detect signals associated with odorant binding to GPCRs has shown promising results. Such artificial nose technology detects and discriminates between odorants they previously “learned” [78]. The combination of such technology with computational biology represents an attractive strategy for improving our knowledge of the molecular mechanisms of these volatile molecules. Information on the olfactory system, the pharmacology profile of individual odorants, the network regulation as well as the pharmacodynamic, toxicological and pharmacokinetic effects is sparse and further investigations must be performed. The pharmacological space of odorant molecules is not exclusively limited to GPCRs (although they are in majority). Direct pathway *via* the ORs, becoming activated by odorants after nasal inhalation in the nose epithelium is well established [79]. Transcellular penetration into the central nervous system by passive diffusion has also been described [80]. Therefore, we could assume that such volatile compounds are not only stimulators of olfactory perception but may be involved in other essential physiological functions related to human health.

## Supporting Information

Figure S1 **Global mapping of the human odorome.** Nodes represent olfactory receptors (ORs) with known binding ligands. Green nodes are human ORs, and blue nodes represent human homologous and orthologous ORs derived from mouse and rat information. The width of the edges correspond to the to the weighted score.
(TIFF)

Figure S2 **Mapping of odorants on the pharmacological space.** Chemical pair-wise similarity network based on the chemical structure and using a Tanimoto coefficient threshold to 0.9. The blue nodes represent compounds with known bioactivity from ChemProt and the green nodes are the odorants from FlavorBase. Edges represent a high structural similarity between two molecules. The edge color indicates the Tanimoto values: orange for Tc between 0.9 and 0.95 and purple for Tc between 0.95 and 1. From such graph, we can assume that an odorant (in green) similar to a compound from ChemProt (in blue) potentially shared the same bioactivity.
(TIFF)

Figure S3 **Protein family distribution.** The values indicate the number of predicted interactions between odorant molecules and proteins. Only families with more than 100 interactions are shown separately, ‘other families’ represent the rest in the graph. This other category contains for example the tyrosinase family, the adenylate kinase family and glycogen phosphorylase family.
(TIFF)

Figure S4 **Concentration-response curves of know ligands of human cannabinoid receptor CB1.** As expected, AEA and HU210 act as agonists whereas AM251 acts as inverse agonist. GloSensor assays were carried out in the absence (•) or in the presence (○) of pertussis toxin-treated cells. Data points and EC50 values are means ± s.e.m. from three experiments.
(TIFF)

File S1 **Combined supporting information file containing Tables S1–S4 and Methods S1.** Table S1: List of sources used to gather odorant molecule-OR interactions. Table S2: List of odor tendencies for the human olfactory receptors used in the OR-OR network. Table S3: Diseases and biological pathways linked to the olfactory system. Table S4: Odorant-OR interactions in Human, Rat and Mouse.
(DOC)
