# Supplementary figures and images for: Identification of Odorant-Receptor Interactions by Global Mapping of the Human Odorome
Source: PLoS One. 2014 Apr 2;9(4):e93037. doi: 10.1371/journal.pone.0093037 (PMC3973694; doi:10.1371/journal.pone.0093037)

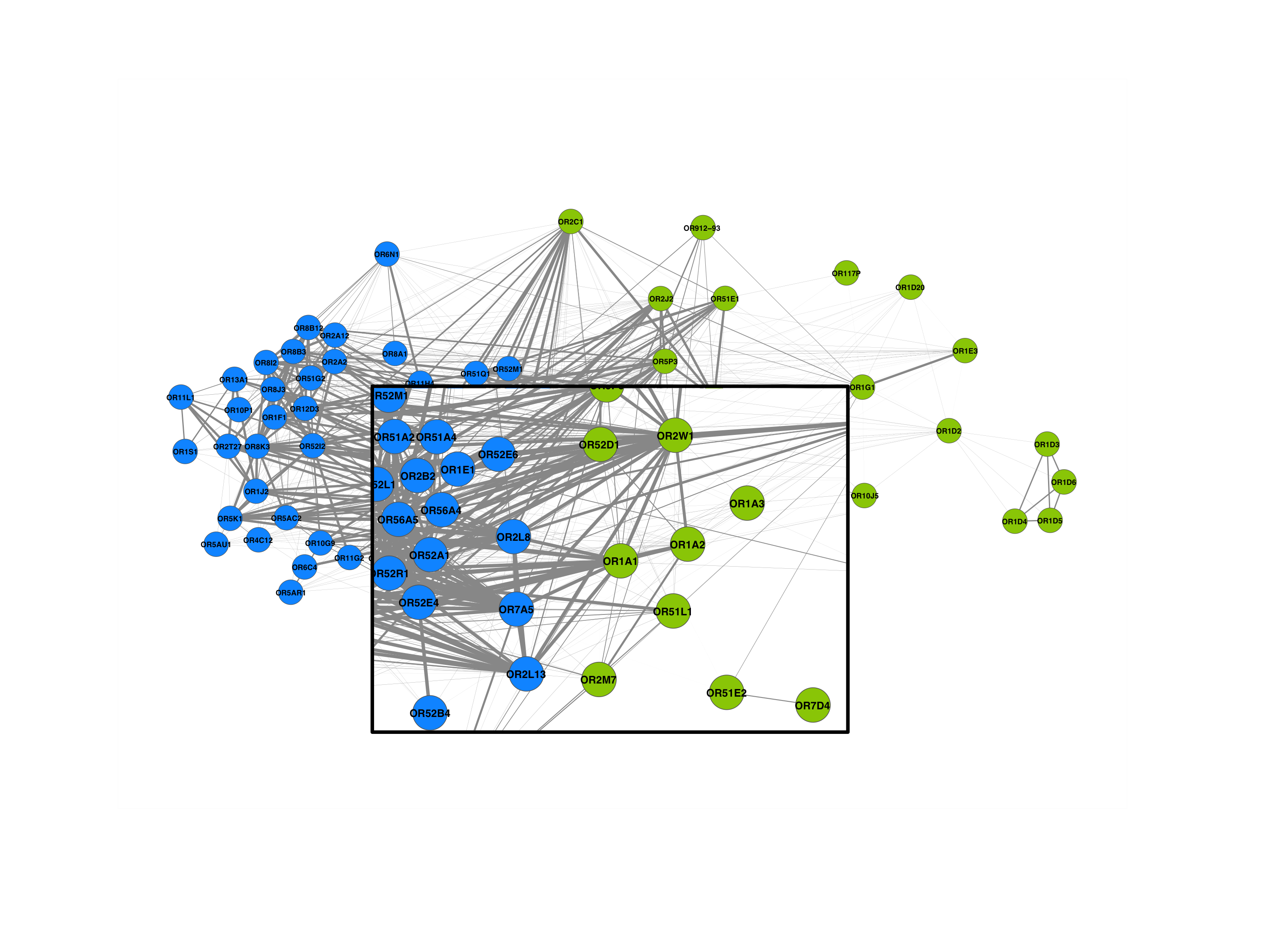

Supplement: Figure S1 — Global mapping of the human odorome. Nodes represent olfactory receptors (ORs) with known binding ligands. Green nodes are human ORs, and blue nodes represent human homologous and orthologous ORs derived from mouse and rat information. The width of the edges correspond to the to the weighted score. (TIFF) [file pone.0093037.s001.tiff]

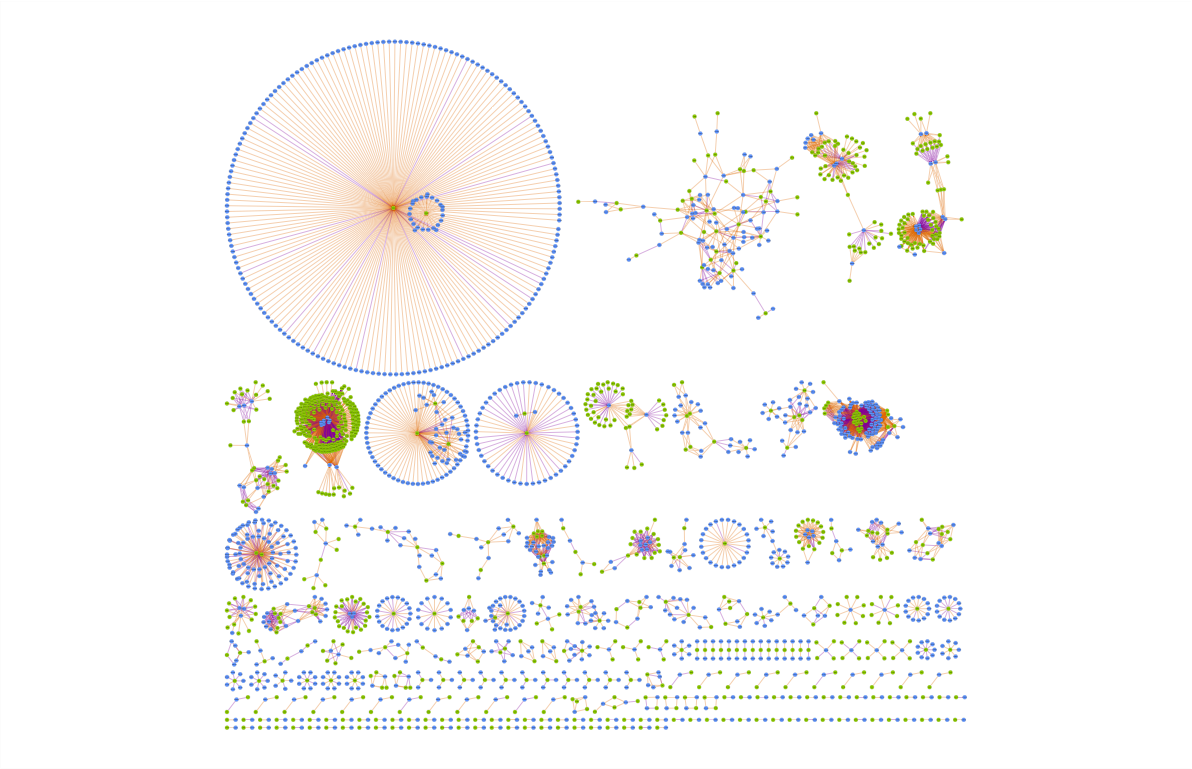

Supplement: Figure S2 — Mapping of odorants on the pharmacological space. Chemical pair-wise similarity network based on the chemical structure and using a Tanimoto coefficient threshold to 0.9. The blue nodes represent compounds with known bioactivity from ChemProt and the green nodes are the odorants from FlavorBase. Edges represent a high structural similarity between two molecules. The edge color indicates the Tanimoto values: orange for Tc between 0.9 and 0.95 and purple for Tc between 0.95 and 1. From such graph, we can assume that an odorant (in green) similar to a compound from ChemProt (in blue) potentially shared the same bioactivity. (TIFF) [file pone.0093037.s002.tiff]

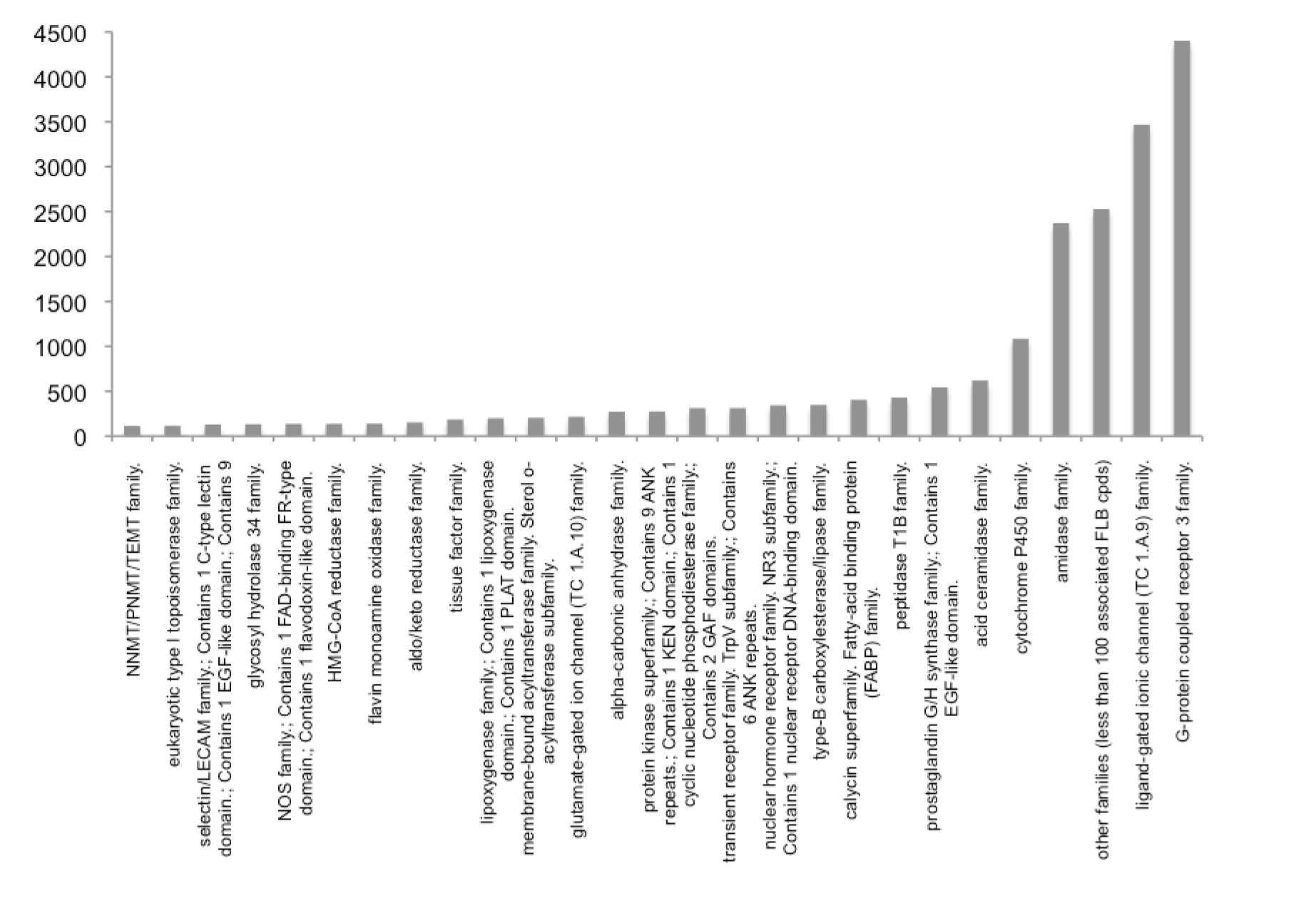

Supplement: Figure S3 — Protein family distribution. The values indicate the number of predicted interactions between odorant molecules and proteins. Only families with more than 100 interactions are shown separately, ‘other families’ represent the rest in the graph. This other category contains for example the tyrosinase family, the adenylate kinase family and glycogen phosphorylase family. (TIFF) [file pone.0093037.s003.tiff]

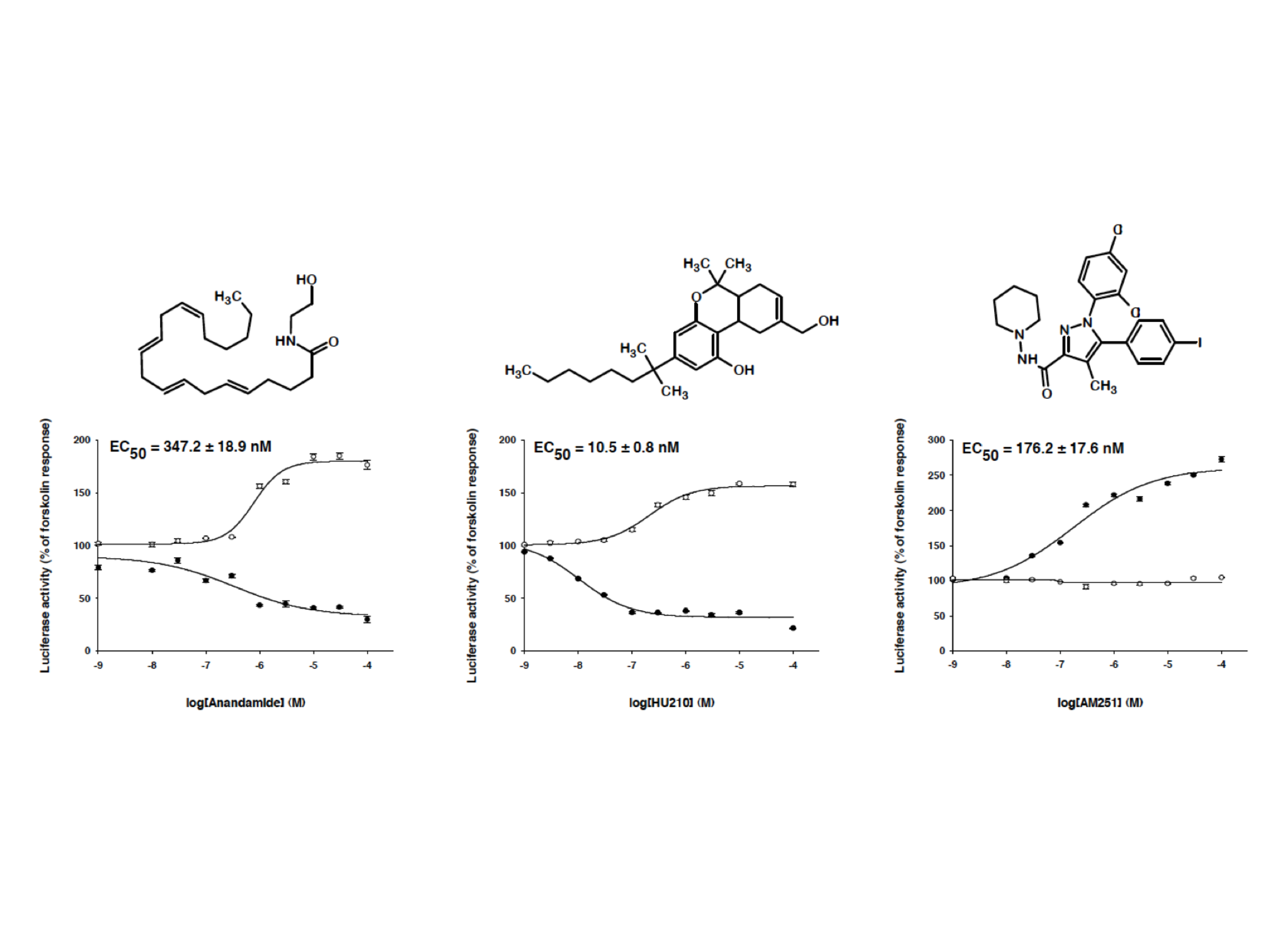

Supplement: Figure S4 — Concentration-response curves of know ligands of human cannabinoid receptor CB1. As expected, AEA and HU210 act as agonists whereas AM251 acts as inverse agonist. GloSensor assays were carried out in the absence (•) or in the presence (○) of pertussis toxin-treated cells. Data points and EC50 values are means ± s.e.m. from three experiments. (TIFF) [file pone.0093037.s004.tiff]
