# Supplementary material for: Identification of Odorant-Receptor Interactions by Global Mapping of the Human Odorome
Source: PLoS One. 2014 Apr 2;9(4):e93037. doi: 10.1371/journal.pone.0093037 (PMC3973694; doi:10.1371/journal.pone.0093037)
Supplement: File S1 — Combined supporting information file containing Tables S1–S4 and Methods S1. Table S1: List of sources used to gather odorant molecule-OR interactions. Table S2: List of odor tendencies for the human olfactory receptors used in the OR-OR network. Table S3: Diseases and biological pathways linked to the olfactory system. Table S4: Odorant-OR interactions in Human, Rat and Mouse. (DOC) [file pone.0093037.s005.doc]

Identification of odorant-receptor interactions by global mapping of the human odorome.

**Supplementary information**

Karine Audouze, Anne Tromelin, Anne Marie Le Bon, Christine Belloir, Rasmus Koefoed Petersen, Karsten Kristiansen, Søren Brunak, Olivier Taboureau.

**SUPPLEMENTARY METHODS**

***GloSensor cAMP assays***

All odorants used in these assays were purchased from Sigma-Aldrich (Saint-Quentin Fallavier, France) except celery ketone, which was kindly provided by B. Schilling (Givaudan Schweiz AG, Dübendorf, Switzerland). The stock solutions of odorants (100 mM) were prepared in dimethylsulfoxide (DMSO) and were stored at -20°C. Immediately prior to the assays, the stock solutions of odorants were diluted in the CO2-independent medium (Gibco®, Life Technologies) to the appropriate concentrations. The final concentration of DMSO in wells never exceeded 1% (v/v); this concentration had no effect on GloSensor cAMP assays.

*Odorant-OR interactions*

To validate predicted odorant compound-OR interactions, we used the GloSensor™ cAMP assay (Promega, Charbonnières-Les-Bains, France) to measure EC_50_ values of compounds. The functional assay was performed in Hana3A cells, a HEK293T-derived cell line optimized for OR expression . Hana3A cells were grown in Minimal Essential Medium (MEM; Life Technologies, Villebon-sur-Yvette, France) supplemented with 10% fetal bovine serum (PAA Laboratories, Les Mureaux, France), 100 μM Eagle’s non-essential amino acids, 2 mM glutamax (Life Technologies) and 1 μg/ml puromycin (Sigma-Aldrich, Saint-Quentin Fallavier, France). The cells were cultured at 37°C in a humidified atmosphere containing 7.3% CO_2_. Forty-eight hours before performing the GloSensor™ cAMP assay, cells were plated on poly-D-lysine coated 96-well plates (BD Biosciences, Le Pont de Claix, France) at a density of 3x10^4^ cells per well. Twenty-four hours later, cells were transiently transfected with the OR expression plasmid (50 ng per well) together with the pCI-RTP1S plasmid and the pGlo™-22F cAMP plasmid (Promega) (10 ng and 100 ng per well, respectively) using Fugene®HD (Promega) according to the manufacturer instructions. In addition, to check that induced activities are OR-dependent, mock transfections were performed by transfecting the cells with the pCI plasmid in place of the OR expression plasmid. Twenty-four hours after transfection, the medium was replaced with a CO_2_-independent medium (Gibco®, Life Technologies) supplemented with the GloSensor™cAMP reagents (Promega) and the plates were incubated for 2 hours at room temperature. Luminescence was measured with a Victor 3V plate reader (Perkin Elmer, Courtaboeuf, France) after addition of odorant solutions (10 μl). A range of concentrations varying from 0.01 μM to 3 mM was tested. Assays were performed in duplicate and each experiment was repeated at least three times. The median effective concentrations (EC_50_ values) were generated using the Sigma Plot software (Systat Software Inc., London, UK). The expression vectors carrying the OR2W1 (pCI-OR2W1), OR51E1 (pCI-OR51E1) and OR5P3 (pCI-OR5P3) genes were kindly provided by Dr. H. Matsunami (Duke University, Durham, USA) through the Addgene repository (http://www.addgene.org). The expression vector carrying the accessory protein RTP1S (pCI-RTP1S) was also provided by Dr. H. Matsunami.

*Odorant-CB1 interactions*

To examine CB1-odorant compound interactions, the GloSensor™ cAMP assay was performed in HEK293T cells obtained from the American Type Cell Culture Collection (LGC Promochem, Molsheim, France). HEK293T cells were plated as described above in Dulbecco’s Modified Eagle Medium (DMEM, Life Technologies) containing 2 mM glutamax and 10% fetal bovine serum. Twenty-four hours before performing assays, cells were transiently co-transfected with the human CB1 plasmid (pcDNA3.1-hCB1) obtained from the Missouri S&T cDNA Resource Center (www.cdna.org) and the pGlo™-22F cAMP plasmid (Promega). In addition, to check that induced activities are CB1-dependent, mock transfections were performed by transfecting the cells with the pcDNA3.1 plasmid in place of the CB1 expression plasmid. To facilitate the detection of Gi/o mediated responses, basal cAMP production was enhanced by pre-incubating cells with 1 μM forskolin (Sigma-Aldrich) for 10 minutes before adding tested compounds. Where indicated, cells were treated with 0.1 μg/ml pertussis toxin (PTX, Sigma-Aldrich) for 24 h before the addition of forskolin. Depending on the compound, a range of concentrations varying from 1 nM to 10 mM was tested. Assays were performed in duplicate and each experiment was repeated three times. Results were expressed as the percentages of forskolin-stimulated luciferase activity. The EC_50_ values were generated using the Sigma Plot software.

Before investigating the effects of predicted compounds on human CB1 activation, we checked that our experimental system worked efficiently by testing cannabinoids acting as agonists (AEA and HU210) or inverse agonist (AM251). The endocannabinoid anandamide (AEA) and the synthetic cannabinoids (6aR)-trans-3-(1,1-dimethylheptyl)-6a,7,10,10a,-tetrahydro-1-hydroxy-6,6-dimethyl-6H-dibenzo[b,d]pyran-9-methanol (HU210) and N-(piperidin-1-yl)-5-(4-iodophenyl)-1-(2,4-dichlorophenyl)-4-methyl-1H-pyrazole-3-carboxamide (AM251) were obtained from Tocris (Tocris Bioscience, Bristol, United Kingdom). The stock solutions of drugs (10 mM) were dissolved in DMSO and stored at -20°C. Immediately prior to the assays, the stock solutions were diluted in the CO2-independent medium to the appropriate concentrations. Functional assays were performed in the absence or presence of pertussis toxin (PTX)-treated cells. PTX blocks the function of the Gi/o protein through an irreversible ADP ribosylation. In agreement with previous studies indicating the functional coupling of the CB1 receptor with Gi/o proteins , we observed that AEA and HU210 elicited a significant reduction in cAMP production in control cells while an increase in cAMP formation was detected in PTX-treated cells (Fig. S4). Conversely, as expected, AM251 strongly stimulated luciferase activity in control cells. This effect was inhibited in PTX-treated cells.

***Competitive PPARγ binding assay***

To validate predictions of odorant-PPARγ interactions, IC50 values for respective compounds were determined by competitive binding using time-resolved fluorescence resonance energy transfer (LanthaScreen, Invitrogen) on a Wallac EnVision (PerkinElmer). A terbium labeled anti-GST antibody was used to label purified GST-tagged human PPARγ ligand binding domain. Energy transfer from terbium to the tracer, a fluorescent pan PPAR agonist, enabled read-out of each test compound’s ability to displace the tracer. RFU values from dose-response curves (10 concentrations: 0.01-300 mM)(duplicate sampling) for test compounds as well as positive control (Rosiglitazone)(10 concentrations: 0.3-10000 nM) were then analyzed using GraphPad Prism (GraphPad Software). An unrestrained sigmoidal (one-binding site) dose-response curve was fitted to each data set by linear regression allowing for determination of IC50 values.

***PPAR-LBD Transactivation***

A mouse embryo fibroblast cell line was used for PPARg transfections. Cells were propagated in Dulbeccos Modified Eagle’s Media supplemented with 10% fetal calf serum and antibiotics. For transfections, cells were transfected in solution by Metafectene lipofection, essentially according to the manufacturer’s (Biontex) instructions and seeded in Dulbeccos Modified Eagle’s Media supplemented with 10% fetal calf serum and antibiotics in 96-well dishes at 24000 cells/cm2. The transfection plasmid mix included the Gal4-responsive luciferase reporter, the expression vector for the fusion between the Gal4 DNA-binding domain and the ligand binding domain of human PPARgamma, and a CMV-Renilla normalization vector. 6 hours after seeding the transfected cells, new media containing the DMSO vehicle (0.1 %), positive control Rosiglitazone (1 mM) or the test compound was added. Approx. 18 hours later, cells were harvested and lysates analyzed for Photinus and Renilla luciferase activity by luminometry. All data points were performed with at least 6 datapoints. Luminometer raw data was analyzed in Microsoft Excel spreadsheets and presented as column graphs depicting average values ± standard deviations.

**SUPPORTING INFORMATION LEGENDS**

Figure S1: Global mapping of the human odorome**.**

Nodes represent olfactory receptors (ORs) with known binding ligands. Green nodes are human ORs, and blue nodes represent human homologous and orthologous ORs derived from mouse and rat information. The width of the edges correspond to the to the weighted score.

Figure S2: Mapping of odorants on the pharmacological space.

Chemical pair-wise similarity network based on the chemical structure and using a Tanimoto coefficient threshold to 0.9. The blue nodes represent compounds with known bioactivity from ChemProt and the green nodes are the odorants from FlavorBase. Edges represent a high structural similarity between two molecules. The edge color indicates the Tanimoto values: orange for Tc between 0.9 and 0.95 and purple for Tc between 0.95 and 1. From such graph, we can assume that an odorant (in green) similar to a compound from ChemProt (in blue) potentially shared the same bioactivity.

Figure S3: Protein family distribution.

The values indicate the number of predicted interactions between odorant molecules and proteins. Only families with more than 100 interactions are shown separately, ‘other families’ represent the rest in the graph. This other category contains for example the tyrosinase family, the adenylate kinase family and glycogen phosphorylase family.

Figure S4: Concentration-response curves of know ligands of human cannabinoid receptor CB1.

As expected, AEA and HU210 act as agonists whereas AM251 acts as inverse agonist. GloSensor assays were carried out in the absence (●) or in the presence (🞅) of pertussis toxin-treated cells. Data points and EC50 values are means ± s.e.m. from three experiments.

**SUPPLEMENTARY TABLES**

**Table S1**. List of sources used to gather odorant molecule-OR interactions.

| Abaffy, T., Malhotra, A. & Luetje, C. W. The molecular basis for ligand specificity in a mouse olfactory receptor - a network of functionally important residues. *J. Biol. Chem*. **282**, 1216-1224 (2007). |
| --- |
| Araneda, R.C., Kini, A.D. & Firestein, S. [The molecular receptive range of an odorant receptor.](http://www.ncbi.nlm.nih.gov/pubmed/11100145) *Nat. Neurosci*. **3**, 1248-1255 (2000). |
| Araneda, R.C., Peterlin, Z., Zhang, X., Chesler, A. & Firestein, S. [A pharmacological profile of the aldehyde receptor repertoire in rat olfactory epithelium.](http://www.ncbi.nlm.nih.gov/pubmed/14724183) *J. Physiol*. **16**, 743-756 (2004). |
| [Baud, O](http://www.ncbi.nlm.nih.gov/pubmed?term="Baud O"%5BAuthor%5D). *et al*. The mouse eugenol odorant receptor: structural and functional plasticity of a broadly tuned odorant binding pocket. [*Biochemistry*](http://www.ncbi.nlm.nih.gov/pubmed?term=baud odor 2011) **50**, 843-853 (2011). |
| [Benilova, I](http://www.ncbi.nlm.nih.gov/pubmed?term="Benilova I"%5BAuthor%5D). *et al*. Stimulation of human olfactory receptor 17-40 with odorants probed by surface plasmon resonance. [*Eur. Biophys. J.*](http://www.ncbi.nlm.nih.gov/pubmed?term=Benilova  2008) **37**, 807-814 (2008). |
| Chen, H., Dadsetan, S., Fomina, A.F. & Gong, Q. Expressing exogenous functional odorant receptors in cultured olfactory sensory neurons. *Neural Dev*. **3**, 22 (2008). |
| Doszczak, L. *et al*. Prediction of perception: Probing the hor17-4 olfactory receptor model with silicon analogues of bourgeonal and lilial. *Angew. Chem. Int. Ed. Engl.* **46**, 3367-3371 (2007). |
| Floriano, W.B., Vaidehi, N. & Goddard, W.A. Making sense of olfaction through predictions of the 3-D structure and function of olfactory receptors, *Chem. Sens.* **29**, 269-290 (2004). |
| Fujita, Y., Takahashi, T., Suzuki, A., Kawashima, K., Nara, F. & Koishi, R. Deorphanization of Dresden G protein-coupled receptor for an odorant receptor. *J. Recept. Signal Transduct. Res*. **27**, 323-334 (2007). |
| Gaillard, I., Rouquier, S., Pin, J.P., Mollard, P., Richard, S., Barnabé, C., Demaille, J. & Giorgi, D. A single olfactory receptor specifically binds a set of odorant molecules. *Eur. J. Neurosci*. **15**, 409-418 (2002). |
| Gaillard, I., Rouquier, S., Chavanieu, A., Mollard, P. & Giorgi, D. Amino-acid changes acquired during evolution by olfactory receptor 912-93 modify the specificity of odorant recognition. *Hum. Mol. Genet.* **13**, 771-780 (2004). |
| Grosmaitre, X., Fuss, S.H., Lee, A.C., Adipietro, K.A., Matsunami, H., Mombaerts, P. & Ma, M. SR1, a mouse odorant receptor with an unusually broad response profile. *J. Neurosci*. **29**, 14545-14552 (2009). |
| Haddad, R., Carmel, L., Sobel, N. & Harel, D. Predicting the receptive range of olfactory receptors. *PLoS Comput. Biol*. **4**, e18 (2008). |
| Hall, S.E., Floriano, W.B., Vaidehi, N. & Goddard, W.A. Predicted 3-D structures for mouse I7 and rat I7 olfactory receptors and comparison of predicted odor recognition profiles with experiment. *Chem. Sens.* **29**, 595-616 (2004). |
| Jacquier, V., Pick, H. & Vogel, H. [Characterization of an extended receptive ligand repertoire of the human olfactory receptor OR17-40 comprising structurally related compounds.](http://www.ncbi.nlm.nih.gov/pubmed/16539658) *J. Neurochem*. **97**, 537-544 (2006). |
| Kajiya, K., Inaki, K., Tanaka, M., Haga, T., Kataoka, H. & Touhara, K. Molecular bases of odor discrimination: Reconstitution of olfactory receptors that recognize overlapping sets of odorants. *J. Neurosci*. **21**, 6018-6025 (2001). |
| Katada, S., Nakagawa, T., Kataoka, H. & Touhara, K. Odorant response assays for a heterologously expressed olfactory receptor. *Biochem. Biophys. Res. Commun*. **305**, 964-969 (2003). |
| Katada, S., Hirokawa, T., Oka, Y., Suwa, M. & Touhara, K. Structural basis for a broad but selective ligand spectrum of a mouse olfactory receptor: Mapping the odorant-binding site. *J. Neurosci.* **25**, 1806-1815 (2005). |
| Katada, S., Hirokawa, T. & Touhara, K. Exploring the odorant binding site of a G-protein-coupled olfactory receptor. *Curr. Comput. Aided Drug Des.* **4**, 123-131 (2008). |
| Kato, A., Katada, S. & Touhara, K. Amino acids involved in conformational dynamics and G protein coupling of an odorant receptor: targeting gain-of-function mutation. *J. Neurochem*. **107**, 1261-1270 (2008). |
| Keller, A., Zhuang, H., Chi, Q., Vosshall, L.B. & Matsunami, H. [Genetic variation in a human odorant receptor alters odour perception.](http://www.ncbi.nlm.nih.gov/pubmed/17873857) *Nature* **449**, 468-472 (2007). |
| Ko, H.J. & Park, T.H. Piezoelectric olfactory biosensor: ligand specificity and  dose-dependence of an olfactory receptor expressed in a heterologous cell system. *Biosens. Bioelectron*. **20**, 1327-1332 (2005). |
| Ko, H.J. & Park, T.H. Dual signal transduction mediated by a single type of  olfactory receptor expressed in a heterologous system. *Biol. Chem*. **387**, 59-68 (2006). |
| Ko, H.J. & Park, T.H. Functional analysis of olfactory receptors expressed in a HEK-293 cell system by using cameleons. *J. Microbiol. Biotechnol*. **17**, 928-933 (2007). |
| [Krautwurst, D](http://www.ncbi.nlm.nih.gov/pubmed?term="Krautwurst D"%5BAuthor%5D)., [Yau, K.W](http://www.ncbi.nlm.nih.gov/pubmed?term="Yau KW"%5BAuthor%5D). & [Reed, R.R](http://www.ncbi.nlm.nih.gov/pubmed?term="Reed RR"%5BAuthor%5D). Identification of ligands for olfactory receptors by functional expression of a receptor library. [*Cell.*](http://www.ncbi.nlm.nih.gov/pubmed?term=krautwurst 1998 odor) **95**, 917-926 (1998). |
| Lai, P.C., Singer, M.S. & Crasto, C.J. Structural activation pathways from dynamic olfactory receptor-odorant interactions. *Chem. Senses* **30**, 781-792 (2005). |
| Li, Y.R. & Matsunami, H. [Activation state of the M3 muscarinic acetylcholine receptor modulates mammalian odorant receptor signaling.](http://www.ncbi.nlm.nih.gov/pubmed/21224444) *Sci. Signal.* **4**, ra1 (2011). |
| [Matarazzo, V](http://www.ncbi.nlm.nih.gov/pubmed?term="Matarazzo V"%5BAuthor%5D). *et al*. Functional characterization of two human olfactory receptors expressed in the baculovirus Sf9 insect cell system. [*Chem. Senses.*](http://www.ncbi.nlm.nih.gov/pubmed?term=matarazzo 2005 odor) **30**, 195-207 (2005). |
| Menashe, I., Abaffy, T., Hasin, Y., Goshen, S., Yahalom, V., Luetje, C.W. & Lancet, D. [Genetic elucidation of human hyperosmia to isovaleric acid.](http://www.ncbi.nlm.nih.gov/pubmed/17973576) *PLoS Biol*. **5**, e284 (2007). |
| Minic, J., Persuy, M.A., Godel, E., Aioun, J., Connerton, I., Salesse, R. & Pajot-Augy, E. Functional expression of olfactory receptors in yeast and development of a bioassay for odorant screening. *FEBS J*. **272**, 524-537 (2005). |
| Neuhaus, E.M., Zhang, W., Gelis, L., Deng, Y., Noldus, J. & Hatt, H. Activation of an olfactory receptor inhibits proliferation of prostate cancer cells. *J. Biol. Chem*. **284**, 16218-16225 (2009). |
| Oka, Y., Nakamura, A., Watanabe, H. & Touhara, K. [An odorant derivative as an antagonist for an olfactory receptor.](http://www.ncbi.nlm.nih.gov/pubmed/15574817) *Chem. Senses* **29**, 815-822 (2004). |
| Oka, Y., Omura, M., Kataoka, H. & Touhara, K. [Olfactory receptor antagonism between odorants.](http://www.ncbi.nlm.nih.gov/pubmed/14685265) *EMBO J.* **23**,120-126. (2004) |
| Peterlin, Z. *et al*. [The importance of odorant conformation to the binding and activation of a representative olfactory receptor.](http://www.ncbi.nlm.nih.gov/pubmed/19101476) *Chem. Biol.* **15**, 1317-1327 (2008). |
| Repicky, S.E. & Luetje, C.W. Molecular receptive range variation among mouse odorant receptors for aliphatic carboxylic acids. *J. Neurochem*. **109**,193-202 (2009). |
| Saito, H., Chi, Q., Zhuang, H., Matsunami, H. & Mainland, J.D. [Odor coding by a Mammalian receptor repertoire.](http://www.ncbi.nlm.nih.gov/pubmed/19261596) *Sci. Signal*. **2**, ra9 (2009). |
| Sanz, G., Schlegel, C., Pernollet, J.C. & Briand, L. [Comparison of odorant specificity of two human olfactory receptors from different phylogenetic classes and evidence for antagonism.](http://www.ncbi.nlm.nih.gov/pubmed/15647465) *Chem. Senses* **30**, 69-80 (2005). |
| Sanz, G., Thomas-Danguin, T., Hamdani, E.H., Le Poupon, C., Briand, L., Pernollet, J.C., Guichard, E. & Tromelin, A. Relationships between molecular structure and perceived odor quality of ligands for a human olfactory receptor. *Chem. Senses* **33**, 639-653 (2008). |
| Schmiedeberg, K., Shirokova, E., Weber, H.P., Schilling, B., Meyerhof, W. & Krautwurst, D. Structural determinants of odorant recognition by the human olfactory receptors or1a1 and or1a2. *J. Struct. Biol*. **159**, 400-412 (2007). |
| Shirokova, E., Schmiedeberg, K., Bedner, P., Niessen, H., Willecke, K., Raguse, J.D., Meyerhof, W. & Krautwurst, D. [Identification of specific ligands for orphan olfactory receptors. G protein-dependent agonism and antagonism of odorants.](http://www.ncbi.nlm.nih.gov/pubmed/15598656) *J. Biol. Chem*. **280**, 11807-11815 (2005). |
| Skoufos, E., Marenco, L., Nadkarni, P.M., Miller, P.L. & Shepherd, G.M. [Olfactory receptor database: a sensory chemoreceptor resource.](http://www.ncbi.nlm.nih.gov/pubmed/10592268) *Nucleic Acids Res*. **28**, 341-343 (2000). |
| Spehr, M,, Gisselmann, G., Poplawski, A., Riffell, J.A., Wetzel, C.H., Zimmer, R.K. & Hatt, H. [Identification of a testicular odorant receptor mediating human sperm chemotaxis.](http://www.ncbi.nlm.nih.gov/pubmed/12663925) *Science* **299**, 2054-2058 (2003). |
| Touhara, K. Deorphanizing vertebrate olfactory receptors: Recent advances in odorant-response assays. *Neurochem. Int*. **51**, 132-139 (2007). |
| Touhara, K., Sengoku, S., Inaki, K., Tsuboi, A., Hirono, J., Sato, T., Sakano, H. & Haga, T. [Functional identification and reconstitution of an odorant receptor in single olfactory neurons.](http://www.ncbi.nlm.nih.gov/pubmed/10097159) *Proc. Natl. Acad. Sci. USA* **96**, 4040-4045 (1999). |
| [Triller, A](http://www.ncbi.nlm.nih.gov/pubmed?term="Triller A"%5BAuthor%5D)., [Boulden, E.A](http://www.ncbi.nlm.nih.gov/pubmed?term="Boulden EA"%5BAuthor%5D)., [Churchill, A](http://www.ncbi.nlm.nih.gov/pubmed?term="Churchill A"%5BAuthor%5D)., [Hatt, H](http://www.ncbi.nlm.nih.gov/pubmed?term="Hatt H"%5BAuthor%5D)., [Englund, J](http://www.ncbi.nlm.nih.gov/pubmed?term="Englund J"%5BAuthor%5D)., [Spehr, M](http://www.ncbi.nlm.nih.gov/pubmed?term="Spehr M"%5BAuthor%5D). & [Sell, C.S](http://www.ncbi.nlm.nih.gov/pubmed?term="Sell CS"%5BAuthor%5D). Odorant-receptor interactions and odor percept: a chemical perspective**.** [*Chem. Biodivers*.](http://www.ncbi.nlm.nih.gov/pubmed?term=triller odor) **5**, 862-886 (2008). |
| Vidic, J.M., Grosclaude, J., Persuy, M.A., Aioun, J., Salesse, R. & Pajot-Augy, E. Quantitative assessment of olfactory receptors activity in immobilized nanosomes: a novel concept for bioelectronic nose. *Lab. Chip*. **6**, 1026-1032 (2006). |
| Vidic, J., Grosclaude, J., Monnerie, R., Persuy, M.A., Badonnel, K., Baly, C., Caillol, M., Briand, L., Salesse, R. & Pajot-Augy, E. [On a chip demonstration of a functional role for Odorant Binding Protein in the preservation of olfactory receptor activity at high odorant concentration.](http://www.ncbi.nlm.nih.gov/pubmed/18432336) *Lab Chip*. **8**, 678-688 (2008). |
| Wetzel, C.H., Oles, M., Wellerdieck, C., Kuczkowiak, M., Gisselmann, G. & Hatt, H. [Specificity and sensitivity of a human olfactory receptor functionally expressed in human embryonic kidney 293 cells and Xenopus Laevis oocytes.](http://www.ncbi.nlm.nih.gov/pubmed/10460249) *J Neurosci*. **19**, 7426-7433 (1999). |
| Zhuang, H., Chien, M.S. & Matsunami, H. *Proc. Natl. Acad. Sci. USA.* **106**, 21247-21251 (2009). |

**Table S2**: List of odor tendencies for the human olfactory receptors used in the OR-OR network. Association score (AS) values are calculated as described in the Methods section in supplementary. The higher is the AS value, the more significant is the association between an OR and an odor perception in human.

| **OR name** | **Odor description** | **AS value** |
| --- | --- | --- |
| OR10J5 | aldehyde | 9.82e-05 |
| OR117P | sour | 0.000147 |
| OR1A1 | sweet | 0.016836 |
|  | fruity | 0.015194 |
|  | floral | 0.012513 |
|  | rose | 0.011065 |
| OR1A2 | sweet | 0.007113 |
|  | floral | 0.006793 |
|  | rose | 0.006547 |
| OR1A3 | fruity | 4.209e-05 |
| OR1D2 | floral | 0.005858 |
|  | light | 0.002651 |
|  | ocean | 0.002357 |
|  | clean | 0.002357 |
| OR1D20 | floral | 0.013865 |
|  | sweet | 0.008249 |
|  | heliotrop | 0.005303 |
|  | green | 0.003928 |
| OR1D3 | powerfull | 9.821e-05 |
|  | muguet | 6.547e-05 |
| OR1D4 | powerfull | 9.821e-05 |
|  | muguet | 6.547e-05 |
| OR1D5 | powerfull | 9.821e-05 |
|  | muguet | 6.547e-05 |
| OR1D6 | powerfull | 9.821e-05 |
|  | muguet | 6.547e-05 |
| OR1E3 | medicine | 0.000196 |
|  | phenol | 0.000196 |
|  | harsh | 0.000117 |
| OR1G1 | fruity | 0.060779 |
|  | sweet | 0.054550 |
|  | fat | 0.030684 |
|  | wax | 0.009958 |
| OR2C1 | butter | 0.000471 |
|  | pineapple | 0.00039 |
|  | sweet | 0.00037 |
|  | fat | 0.00037 |
| OR2J2 | sweet | 0.00037 |
|  | orange | 0.000336 |
|  | oil | 0.000294 |
| OR2M7 | rose | 0.000261 |
|  | sweet | 0.000168 |
|  | floral | 0.000138 |
| OR2W1 | fruity | 0.008249 |
|  | fat | 0.007113 |
|  | sweet | 0.005093 |
|  | rose | 0.002357 |
| OR51E1 | odorless | 0.009428 |
|  | cheese | 0.002887 |
|  | sour | 0.002357 |
| OR51E2 | sour.milk | 0.000196 |
| OR51L1 | pineapple | 9.821e-05 |
|  | honey | 9.821e-05 |
| OR52D1 | fruity | 0.045837 |
|  | sweet | 0.045837 |
|  | fat | 0.018562 |
|  | strong | 0.017727 |
| OR5P3 | fat | 0.002693 |
|  | sherry | 0.002357 |
|  | caraway | 0.002357 |
| OR912-93 | fruity | 0.000168 |
|  | blue.cheese | 0.000117 |

**Table S3**: List of diseases and biological pathways predicted to be linked to the olfactory system using GeneCards (adjusted *p*-value), KEGG pathways (adjusted *p*-value) and Reactome pathways (adjusted *p*-value). The genes, listed by EntrezGeneIDs, are connected to disease or pathway in a source. The source corresponds to the database used for the prediction.

| **DISEASE /PATHWAY** | **NUMBER OF GENES** | **GENES** | **SOURCE** | **P-VAL** |
| --- | --- | --- | --- | --- |
| hypertension | 13 | ADORA2A;ADRB1;ADRB2;ADRB3;ADRBK1;DRD1;DRD5;F2R;GCGR;GNAI1;GNAS;GNB3;CACNA1B | GeneCards | 5.09e-5 |
| schizophrenia | 19 | CHRM1;CHRM5;CNP;ADORA2A;DRD1;DRD5;F2R;GNAI1;GNAL;GNB3;HRH2;HTR4;HTR6;HTR7;OPRK1;OPRM1;PDYN;PIK3CG;CACNA1A | GeneCards | 7.12e-5 |
| mood disorders | 9 | ADRB2;DRD1;PLCB1;GNAI1;GNAL;GNB3;HTR6;PDYN;POMC | GeneCards | 0.0025 |
| calcium signaling pathway | 34 | ADCY1;ADCY2;ADCY3;CHRM1;ADCY7;CHRM3;CHRM5;ADCY8;ADCY9;ADORA2A;ADORA2B;ADRB1;ADRB2;ADRB3;DRD1;ADCY4;F2R;PLCB1;GNA15;GNAL;GNAS;HRH2;HTR4;HTR5A;HTR6;HTR7;PLCE1;PLCB2;PLCB3;PLCB4;CACNA1A;CACNA1B;CACNA1C;GNA14 | KEGG | 1.60e-24 |
| neuroactive ligand-receptor interaction pathway | 23 | CHRM1;CHRM3;CHRM5;ADORA2A;ADORA2B;ADRB1;ADRB2;ADRB3;DRD1;DRD5;F2R;F2RL2;GCGR;HRH2;HTR4;HTR5A;HTR6;HTR7;MTNR1A;MTNR1B;OPRK1;OPRM1;F2RL3 | KEGG | 3.50e-8 |
| taste transduction pathway | 12 | ADCY6;ADCY8;ADCY4;GNAS;GNB1;GNB3;GNG3;GNAT3;GNG13;PLCB2;CACNA1A;CACNA1B | KEGG | 3.60e-8 |
| type 2 diabetes pathway | 11 | PIK3R5;PIK3CA;PIK3CB;PIK3CD;PIK3CG;PKLR;TNF;CACNA1A;CACNA1B;CACNA1C;PIK3R3 | KEGG | 6.91.e-8 |
| long term depression pathway | 9 | GNA13;PLCB1;GNAI1;GNAI3;GNAS;PLCB2;PLCB3;PLCB4;CACNA1A | KEGG | 0.002 |
| opioid signaling pathway | 26 | ADCY1;ADCY2;ADCY3;ADCY5;ADCY6;ADCY7;ADCY8;ADCY9;ADRBK1;ADCY4;PLCB1;GNA15;GNAI1;GNAI3;GNAL;GNAT1;GNAT2;GNAT3;OPRM1;PDYN;PLCB2;PLCB3;PLCB4;GNG2;POMC;GNA14 | Reactome | 1.90e-24 |
| integration of energy metabolism | 32 | ADCY1;ADCY2;ADCY3;ADCY5;ADCY6;ADCY7;ADCY8;ADCY9;CYC1;ADCY4;GCG;GCGR;GNAS;GNB1;GNB2;GNB3;GNG3;GNG4;GNG5;GNG7;GNG10;GNG11;NDUFB2;GNG13;PKLR;GNG2;PRKAG1;GNG12;GNB4;CACNA1A;CACNA1C;GNG8 | Reactome | 2.13e-16 |
| signaling by GPCR pathway | 40 | CHRM1;CHRM3;CHRM5;ADORA2A;ADORA2B;ADRB1;ADRB2;ADRB3;DRD1;DRD5;F2R;F2RL2;GCG;GCGR;GNA15;GNAI1;GNAI3;GNAL;GNAS;GNAT1;GNAT2;GNB1;GNGT1;HRH2;HTR4;HTR5A;HTR6;HTR7;GNAT3;MTNR1A;MTNR1B;OPRK1;OPRM1;PDYN;GNG2;POMC;OPN1LW;RHO;F2RL3;GNA14 | Reactome | 2.46e-7 |

**Table S4**: Data statistics: unique chemical, unique protein and chemical-protein interactions at the different steps of the procedure.

|  | **Statistics** | | |
| --- | --- | --- | --- |
|  | **Chemical** | **Protein** | **Interaction*** |
| Human | 189 | 24 | 463 |
| Rat (ortholog) | 96 | 10 | 170 |
| Mouse (ortholog and homolog) | 174 | 63 | 605 |
| All unique | 323 | 83 | 938 |

* Curated relationships from literature and database. The used sources are listed on Table S1.

**References**

1. Saito H, Kubota M, Roberts RW, Chi QY, Matsunami H (2004) RTP family members induce functional expression of mammalian odorant receptors. Cell 119: 679-691.

2. Felder CC, Joyce KE, Briley EM, Mansouri J, Mackie K, et al. (1995) Comparison of the pharmacology and signal-transduction of the human cannabinoid CB1 and CB2 receptors. Mol Pharmacol 48: 443-450.
